# The Three Catecholics Benserazide, Catechol and Pyrogallol are GPR35 Agonists

**DOI:** 10.3390/ph6040500

**Published:** 2013-04-08

**Authors:** Huayun Deng, Ye Fang

**Affiliations:** Biochemical Technologies, Science and Technology Division, Corning Inc., Corning, NY 14831, USA; E-Mail: dengh@corning.com

**Keywords:** GPR35, benserazide, catechol, pyrogallol

## Abstract

Nearly 1% of all clinically used drugs are catecholics, a family of catechol-containing compounds. Using label-free dynamic mass redistribution and Tango β-arrestin translocation assays, we show that several catecholics, including benserazide, catechol, 3-methoxycatechol, pyrogallol, (+)-taxifolin and fenoldopam, display agonistic activity against GPR35.

## 1. Introduction

Identification of the hidden mechanisms of action of drugs and natural products is essential to elucidate their *in vivo* effects and for drug repurposing. Catecholics, a family of catechol-containing compounds, is rich in natural products as well as in clinically used drugs. Catecholics are well known for their ability to protect against lipid peroxidation by non-enzymatic scavenging of free radicals with their catechol moiety. Furthermore, a recent study showed that out of the 8,659 drugs recorded in the Comprehensive Medicinal Chemistry database 78 are catecholics, and 17 are prescribed by the US Food & Drug Administration [1].

The natural agonists for GPR35, a poorly characterized G protein-coupled receptor (GPCR), remain controversial. Several candidate agonists have been proposed to be the natural agonists including kynurenic acid [2], 2-acyllysophosphatidic acid [3], 5,6-dihydroxyindole-2-carboxylic acid (DHICA) [4], and cyclic guanosine monophosphate (cGMP) [5]. Recent efforts in elucidating the biology and pathophysiology of GPR35 have shown that GPR35 has a relatively promiscuous activation profile, in that several classes of structurally distinct chemicals have been found to be surrogate agonists with generally low potency for the receptor [6,7,8,9,10,11,12,13,14,15]. Among these identified surrogate agonists, several classes of GPR35 agonists contain a negatively charged functional group such as a carboxylic acid, which appears to be an essential moiety for interacting with and activating the GPR35 [2,8,10]. However, some known GPR35 agonists are phenolic drugs including entacapone [9], nitecapone and tolcapone [15], as well as natural phenols including quercetin [8,11], ellagic acid and 7-deshydroxypyrogallin-4-carboxylic acid [11], gallic acid and wedelolactone [13], levodopa and rosmarinate [4], and DHICA [4,14]. Structure-activity relationship analysis of these phenolic agonists led us to postulate and confirm that catechol and its derivatives without carboxylic acid moiety are GPR35 agonists too.

## 2. Experimental

Benserazide, (−)-catechin, catechol, 3,4-dimethoxycinnamic acid, fenoldopam HBr, gallic acid, 3-methoxycatechol, pyrogallol, propyl gallate, syringic acid, and (−)-taxifolin were obtained from Sigma Chemical Co. (St. Louis, MO, USA). Zaprinast and ML145 were obtained from Tocris Chemical Co. (St. Louis, MO, USA).

Human colorectal adenocarcinoma HT-29 cell line was obtained from American Type Cell Culture (Manassas, VA, USA) and was cultured in McCoy’s 5A Medium (#16600-082, Life Technologies, Grand Island, NY, USA) supplemented with 10% fetal bovine serum (FBS), 4.5g/L glucose, 2 mM glutamine, and antibiotics at 37 °C under air/5% CO_2_. Tango™ U2OS-GPR35-*bla* cells were purchased from Life Technologies and were cultured using McCoy’s 5A medium supplemented with 10% dialyzed FBS, 0.1 μM NEAA, 25 μM Hepes (pH 7.3), 1 mM sodium pyruvate, 100 U/mL penicillin, 100 μg/mL streptomycin, 200 μg/mL zeocin, 50 μg/mL hygromycin, and 100 μg/mL geneticin at 37 °C under 5% CO_2_.

DMR assays were performed using an Epic^®^ system (Corning Inc., Corning, NY, USA). This system records a ligand-induced dynamic mass redistribution (DMR) signal of live cells as a shift in resonant wavelength (in picometer, pm) of a resonant waveguide grating biosensor. The DMR signal obtained is a real-time kinetic response that provides a holistic view of ligand-receptor interaction(s) and its functional consequence in the cells [16,17,18]. For HT-29 cells 30,000 cells per well were seeded into 384well Epic^®^ biosensor plates (Corning) and cultured overnight. For U2OS and U2OS-GPR35-*bla* cells 18,000 cells per well were seeded and cultured overnight. These cells were then washed twice using a plate washer, maintained with Hank’s balanced salt solution (1 × HBSS) and further incubated inside the Epic^®^ system for 1 h. DMR agonist profiling proceeds with a 2-min baseline, followed by compound addition and monitoring the compound-induced responses for about 1 h. DMR antagonist assay proceeds with a 10min preincubation with a GPR35 antagonist (*i.e.*, ML145 [19]), followed by recording a 2min baseline, adding an agonist at a fixed dose (typically at its EC_80_), and recording the cellular response. All DMR signals were background corrected.

Tango assays were performed in engineered U2OS-GPR35-*bla* cell line. This cell line permits an endpoint measurement of the activity of agonists specific to the GPR35 activation-induced β-arrestin translocation [9,10,11]. Specifically, 10000 U2OS-GPR35-*bla* cells per well were seeded in 384-well, black-wall, clear bottom assay plates with low fluorescence background (Corning). After overnight culture, the cells were stimulated with ligands for 5 h at 37 °C under 5% CO_2_, and then loaded with the cell permeable LiveBLAzer™ fluorescence resonance energy transfer (FRET) B/G substrate. After 2 h incubation the coumarin to fluorescein ratio was measured using Victor 4 plate reader (PerkinElmer, Waltham, MA, USA). Results obtained were normalized to the maximal response of zaprinast obtained within the same plate. The maximal response of zaprinast was set to be 100%.

## 3. Results and Discussion

**Figure 1:**
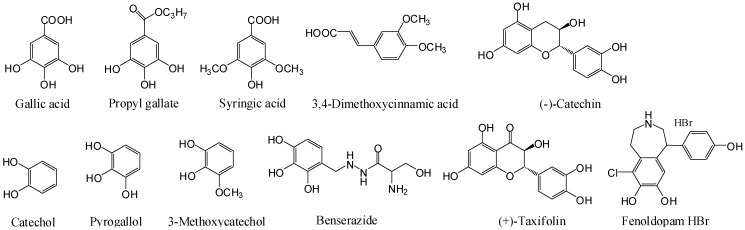
Structures of catecholics examined.

We performed literature mining to identify important catecholics, particularly drugs used clinically and food additives. This led to identification of benserazide and fenoldopam, the two catecholic drugs from DrugBank [20], and propyl gallate the food additive antioxidant [1], beside several catecholics that are known to be GPR35 agonists. Benserazide is a peripherally-acting aromatic L-amino acid decarboxylase or DOPA decarboxylase inhibitor which is used in combination with levodopa for the management of Parkinson’s disease [21]. Fenoldopam is a selective dopamine D_1_ receptor partial agonist used as an antihypertensive agent [22]. Propyl gallate is a food additive (E310, European Food Safety Authority) used to protect oils and fats in products from oxidation. Propyl gallate was recently identified to be an estrogen antagonist [23]. For comparative studies we also tested syringic acid, 3,4-dimethoxycinnamic acid, catechol, 3-methocatechol, pyrogallol, (−)-catechin, and (+)-taxifolin (Figure 1). Gallic acid, zaprinast and YE210 were used as positive controls.

**Figure 2:**
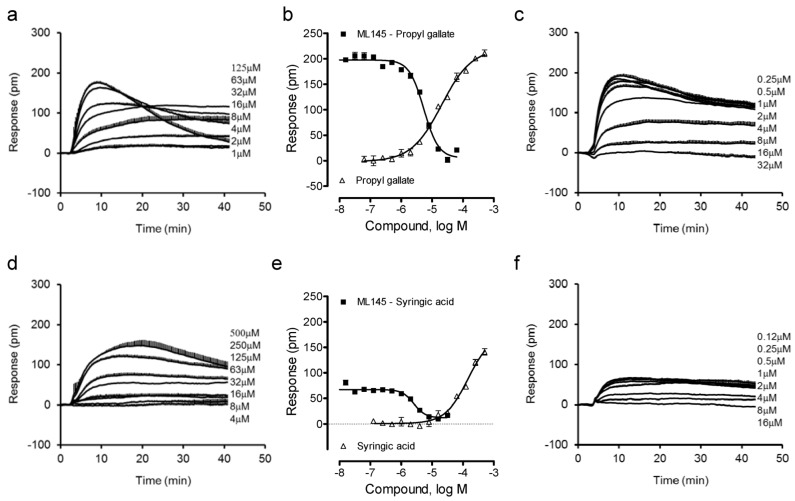
The DMR of gallic acid and caffeic acid analogues in HT-29 cells. (**a**) The DMR dose responses of propyl gallate. (**b**) The maximal DMR amplitude of propyl gallate as a function of its doses, in comparison with the DMR amplitudes of 64 μM propyl gallate as a function of ML1-145 doses. (**c**) The DMR of propyl gallate after pretreatment with ML-145 at different doses. (**d**) The DMR dose responses of syringic acid. (**e**) The maximal DMR amplitude of syringic acid as a function of its doses, in comparison with the DMR amplitude of 200 μM syringic acid as a function of ML1-145 doses. (**f**) The DMR of 100 μM syringic acid after pretreatment with ML-145 at different doses. The data represents mean ± S.D. from two independent measurements (n = 4).

We combined label-free DMR assays in both native HT-29 and engineered U2OS-GPR35-*bla* cells with Tango β-arrestin translocation gene reporter assay in U2OS-GPR35-*bla* cells to ascertain the agonist activity and specificity at the GPR35 of these catecholics. Previously we found that gallic acid is a moderately potent GPR35 agonist with an EC_50_ of 1.16 μM to trigger DMR in HT-29 cells, but caffeic acid is a weak partial agonist [13]. Thus, we first profiled gallic acid and caffeic acid analogues. Results showed that the ester analogue of gallic acid, propyl gallate, still triggered a dose-dependent DMR in HT-29 cells (Figure 2a), yielding a single EC_50_ of 20.2 ± 1.9 μM (n = 4) (Figure 2b). The propyl gallate-induced DMR was dose-dependently and completely blocked by ML-145 (Figure 2c). Similarly, syringic acid, a 3,5-dimethoxy analogue of gallic acid, also triggered a dose-dependent DMR (Figure 2d), but with relatively low potency (EC_50_ of 147 ± 23 μM, n = 4) and efficacy (Figure 2e). ML-145 also dose-dependently and completely blocked the syringic acid DMR (Figure 2f). Furthermore, the 3,4-dimethoxyl analogue of caffeic acid, 3,4-dimethoxycinnamic acid, was found to be inactive. Together, these results suggest that beside the carboxylic acid group the pyrogallol or catechol group is also important to the agonistic activity at the GPR35 of these catecholics, and catechol may be the minimal fragment of these catecholics to activate the GPR35.

**Figure 3:**
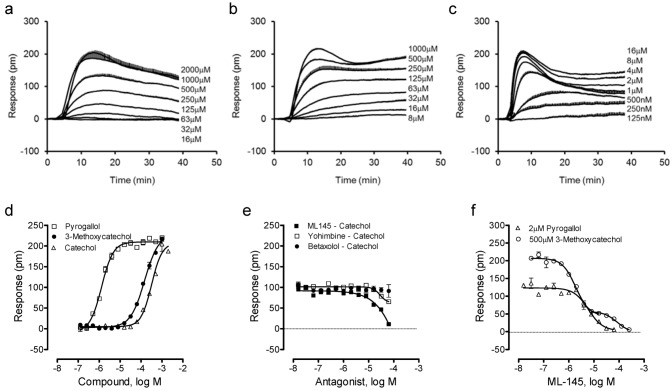
The DMR of catechol analogues in HT-29. (**a**–**c**) Real-time DMR dose responses of catechol (**a**), 3-methoxycatechol (**b**) and pyrogallol (**c**). (**d**) The maximal DMR amplitudes of three catecholics as a function of their doses. (**e**) The DMR amplitude of 500 μM catechol as a function of the doses of three antagonists. (**f**) The DMR amplitudes of 2 μM pyrogallol, or 500 μM 3-methoxycatechol as a function of ML-145 doses. The data represents mean ± S.D. from two independent measurements (n = 4).

We then examined such a possibility by profiling catechol and its analogues using the DMR assay. Results showed that catechol, 3-methoxycatechol and pyrogallol all triggered a dose-dependent DMR in HT-29 (Figure 3a–c), whose characteristics were similar to those induced by other known GPR35 agonists including zaprinast, tyrphostin-51 and YE210 [9,10]. The three catecholics displayed distinct potency, with an EC_50_ rank order of pyrogallol (1.3 ± 0.1 μM) > 3-methoxycatechol (147 ± 15 μM) > catechol (319 ± 27 μM) (n = 4). However, the three catecholics displayed similar efficacy, as evidenced by similar maximal DMR signals (Figure 3d). The antagonist blockage experiments showed that ML-145 completely blocked the catechol DMR, but neither the β-adrenergic receptor antagonist betaxolol nor α_2_-adrenergic receptor antagonist yohimbine had any obvious effects (Figure 3e). HT-29 is known to endogenously express both α_2A_- and β_2_-adrenergic receptors [24,25]; catechol is a partial agonist of β_2_-adrenergic receptor [26]. ML-145 gave rise to a monophasic inhibition of the pyrogallol-induced DMR (IC_50_ of 6.0 ± 0.5 μM, n = 4), but a biphasic inhibition of the 3-methoxycatechol-induced DMR (IC_50_ of 1.9 ± 0.2 and 84.9 ± 7.7 μM, respectively; n = 4) (Figure 3f). One possibility accounted for such distinct inhibition profiles of ML-145 is that the binding site between catecholics and ML-145 is not entirely overlapped [26]; such a possibility is currently under investigation. Alternatively, such distinct inhibitor profiles may be originated from the varying modes of action of ML-145 to block the responses arising from the activation of GPR35 by different agonists [27]. Nonetheless, these results suggest that all three catecholics are GPR35 agonists.

**Figure 4:**
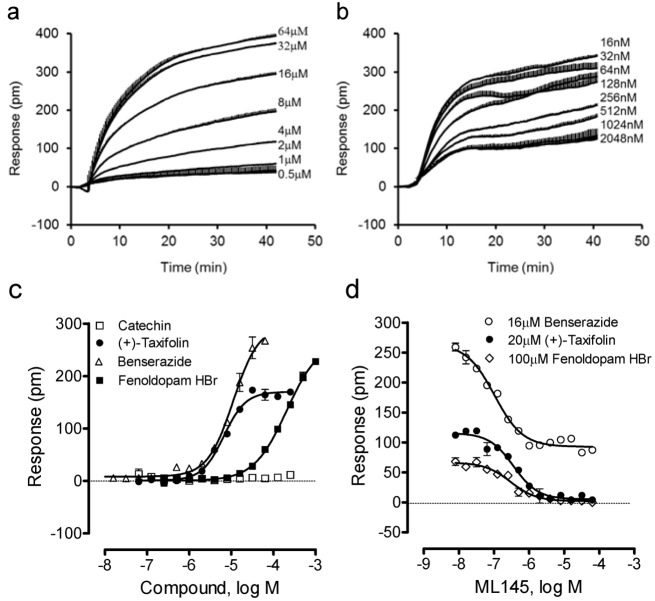
The DMR of four other catecholics in HT-29. (**a**) The DMR dose responses of benserazide. (**b**) The DMR responses of 16 μM benserazide after pretreatment with ML-145 at different doses. (**c**) The maximal DMR amplitudes of four catecholics as a function of their doses. (**d**) The DMR amplitude of 16 μM benserazide, 20 μM (+)-taxifolin, or 100 μM fenoldopam as a function of ML-145 doses. The data represents mean ± S.D. from two independent measurements (n = 4).

We further examined four other catecholics including benserazide, fenoldopam, (−)-catechin and (+)-taxifolin. Results showed that (−)-catechin was inactive, while all other catecholics are active in HT-29 (Figure 4). The potency rank order was (+)-taxifolin (6.6 ± 0.5 μM) > benserazide (11.0 ± 0.9 μM) > fenoldopam (206 ± 19 μM) (n = 4). Interestingly, only benserazide triggered a DMR whose characteristics are distinct from other catecholics (Figure 4a). Blockage experiments showed that ML-145 only partially blocked the DMR of 16 μM benserazide (Figure 4b), but completely blocked the DMR of (+)-taxifolin or fenoldopam (Figure 4d). Given that the DMR of a ligand is a holistic view of its action in the cells and many ligands display polypharmacology [17,18], these results suggest that benserazide also activates another target in HT-29, beside GPR35.

**Figure 5:**
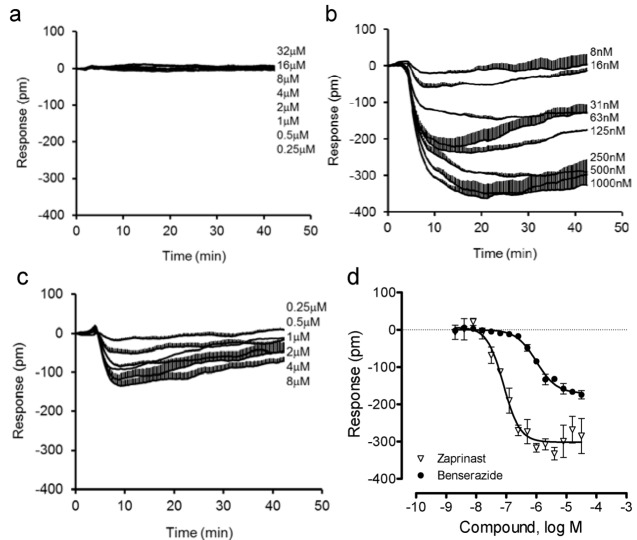
The DMR of zaprinast and benserazide in the parental and engineered U2OS cells. (**a**) The DMR dose responses of benserazide in the parental U2OS cells; (**b**–**d**) the DMR dose responses in U2OS-GPR35-*bla* cells: the real-time DMR of zaprinast (**b**) or benserazide (**c**), and their DMR amplitudes at 8min poststimulation (**d**). The data represents mean ± S.D. from two independent measurements (n = 4).

Next, we compared the DMR of benserazide in U2OS-GPR35-*bla* cells with that in the parental U2OS cells. Results showed that both benserazide and zaprinast were inactive in the parental cells (Figure 5a). In contrast, both ligands triggered a similar negative DMR in the GPR35 expressing cells (Figure 5b,c). Interestingly, compared to zaprinast, benserazide led to a smaller DMR with a lower potency (Figure 5d). The EC_50_ was found to be 80.8 ± 7.1 nM and 1.00 ± 0.13 μM (n = 4) for zaprinast and benserazide, respectively. These results further confirmed that benserazide is a GPR35 agonist.

**Figure 6:**
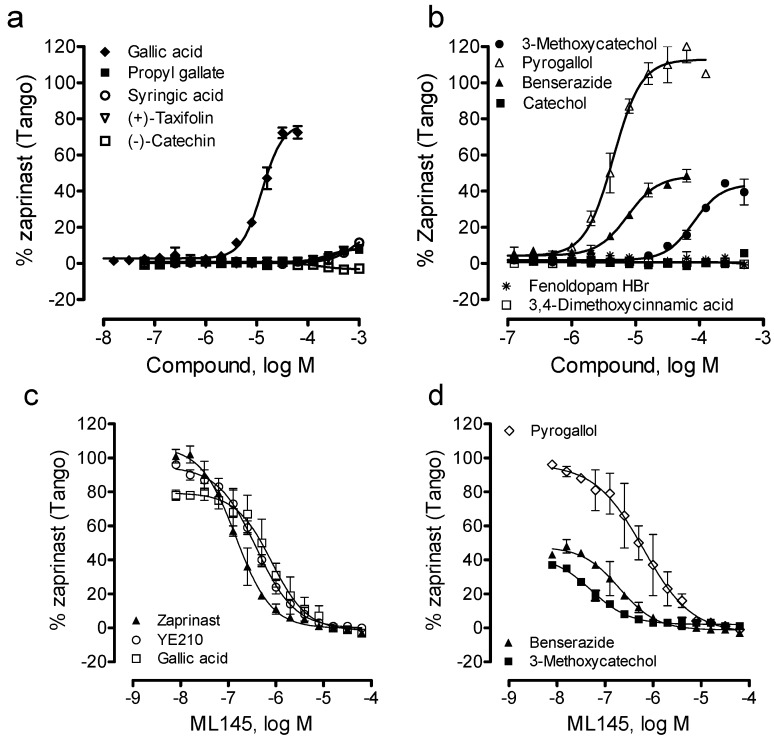
The β-arrestin translocation Tango signals of catecholics in U2OS-GPR35-*bla* cells. (**a**, **b**) The Tango dose responses of catecholics; (**c**, **d**) The ML-145 dose-dependent inhibition profiles of GPR35 agonists at a fixed dose: 10 μM zaprinast, 10 μM YE210, 50 μM gallic acid, 10 μM pyrogallol, 32 μM benserazide, or 250 μM 3-methoxycatechol. The data represents mean ± S.D. from two independent measurements (n = 4).

Lastly, we examined the ability of these catecholics to cause β-arrestin translocation in U2OS-GPR35-*bla* cells using Tango assay. Results showed that among all catecholics examined catechol, (−)-catechin, fenoldopam and 3,4-dimethoxycinnamic acid up to 1mM were inactive; (+)-taxifolin, propyl gallate and syringic acid were weak; and pyrogallol, benserazide and 3-methoxycatechol were active (Figure 6a,b). The potency rank order was pyrogallol (EC_50_, 4.5 ± 0.4 μM) > benserazide (7.5 ± 0.6 μM) > gallic acid (12.6 ± 1.0 μM) > 3-methoxycatechol (80.7 ± 6.3 μM) (n = 4). Compared to DMR assay results, gallic acid displayed much lower potency to cause β-arrestin translocation, while pyrogallol, benserazide and 3-methoxycatechol all gave rise to comparable potency (Table 1). Furthermore, the efficacy rank order was pyrogallol (113 ± 5% of the maximal response of zaprinast) > gallic acid (77 ± 5%) > benserazide (48 ± 4%) ~ 3-methoxycatechol (41 ± 3%). Furthermore, similar to gallic acid, YE210 and zaprinast, ML-145 dose-dependently blocked the Tango signals of three catecholics including pyrogallol, benserazide and 3-methoxycatechol. Together, these results suggest that these β-arrestin translocation-active catecholics are GPR35 agonists.

**Table 1:** Compounds and their pharmacological characteristics. The EC_50_ was obtained using DMR agonism and Tango assays. The data represents mean ± S.D. from 2 independent measurements (n = 4).

Compound	Potency (μM)		Efficacy	
	EC_50, DMR_	EC_50, Tango_	DMR (pm)	Tango (%zaprinast)
Gallic acid	1.16 ± 0.09	12.6 ± 1.0	282	77 ± 5
Propyl gallate	20.2 ± 1.9	weak	210	>8
Syringic acid	147 ± 23	weak	151	>10
Catechol	319 ± 27	inactive	207	0
Pyrogallol	1.3 ± 0.1	4.5 ± 0.4	210	113 ± 5
3-Methoxycatechol	147 ± 15	80.7 ± 6.3	219	41 ± 3
Benserazide	11.0 ± 0.9	7.5 ± 0.6	296	48 ± 4
(+)-Taxifolin	6.6 ± 0.5	weak	170	>8
Fenoldopam	206 ± 19	inactive	268	0
(−)-Catechin	inactive	inactive		
3,4-Dimethoxycinnamic acid	inactive	inactive		

## 4. Conclusions

In summary, we hypothesized and confirmed that the catechol group alone can interact with and activate the GPR35 receptor. This is significant from three different perspectives. First, comparing to gallic acid, pyrogallol displayed much higher potency and efficacy to trigger β-arrestin translocation in U2OS-GPR35-*bla*, but almost identical potency and efficacy to trigger DMR in native HT-29. This suggests that the pyrogallol group may interact with GPR35 via a site that is distinct from the site of interaction of the negatively charged carboxylic group; such a possibility is currently under investigation. Second, the agonistic activity at the GPR35 is a commonly observed property of drugs used for treating Parkinson disease; these drugs include entacapone [9], tolcapone and nitecapone [15], L-DOPA [4], and benserazide (this study). A recent study suggests that entacapone, tolcapone, pyrogallol, gallic acid, caffeic acid, and quercetin all can block fibril formation of α-synuclein and β-amyloid and thus protect against amyloid-induced toxicity [28]. Although the mechanism behind is largely unknown, all these compounds are GPR35 agonists, suggesting a possible role of GPR35 in Parkinson’s disease and other neuronal disorders, which is currently under investigation. Third, we had showed previously that the DMR in HT-29 is mostly sensitive to receptor activity associated with low receptor occupancy state, while the β-arrestin translocation signal in U2OS-GPR35-*bla* cells appears to be biased toward receptor activity associated with high receptor occupancy state [15]. As a matter of fact, the majority of GPR35 agonists examined gave rise to a potency to mediate the DMR in HT-29 that is much higher than its respective potency to trigger β-arrestin translocation in U2OS-GPR35-*bla* cells [4,9,10,11,12,13,14,15]. However, our present results showed that catechol, pyrogallol, 3-methoxycatechol and benserazide, the four analogues without any carboxylic acid moiety, all displayed comparable potency in both types of assays, suggesting that the mechanism for these catechols to active GPR35 may be quite unique.
